# Secure Data Aggregation Using Authentication and Authorization for Privacy Preservation in Wireless Sensor Networks

**DOI:** 10.3390/s24072090

**Published:** 2024-03-25

**Authors:** Samuel Kofi Erskine

**Affiliations:** 1 College of Science and Technology, University of Bridgeport, Bridgeport, CT 06604, USA; serskine@my.bridgeport.edu; 2 Department of Computer Information Science, Florida A & M University, Tallahassee, FL 32310, USA

**Keywords:** wireless sensor networks, secure data aggregation, authentication, authorization, time complexity, energy efficiency, privacy

## Abstract

Existing secure data aggregation protocols are weaker to eliminate data redundancy and protect wireless sensor networks (WSNs). Only some existing approaches have solved this singular issue when aggregating data. However, there is a need for a multi-featured protocol to handle the multiple problems of data aggregation, such as energy efficiency, authentication, authorization, and maintaining the security of the network. Looking at the significant demand for multi-featured data aggregation protocol, we propose secure data aggregation using authentication and authorization (SDAAA) protocol to detect malicious attacks, particularly cyberattacks such as sybil and sinkhole, to extend network performance. These attacks are more complex to address through existing cryptographic protocols. The proposed SDAAA protocol comprises a node authorization algorithm that permits legitimate nodes to communicate within the network. This SDAAA protocol’s methods help improve the quality of service (QoS) parameters. Furthermore, we introduce a mathematical model to improve accuracy, energy efficiency, data freshness, authorization, and authentication. Finally, our protocol is tested in an intelligent healthcare WSN patient-monitoring application scenario and verified using an OMNET++ simulator. Based upon the results, we confirm that our proposed SDAAA protocol attains a throughput of 444 kbs, representing a 98% of data/network channel capacity rate; an energy consumption of 2.6 joules, representing 99% network energy efficiency; an effected network of 2.45, representing 99.5% achieved overall performance of the network; and time complexity of 0.08 s, representing 98.5% efficiency of the proposed SDAAA approach. By contrast, contending protocols such as SD, EEHA, HAS, IIF, and RHC have throughput ranges between 415–443, representing 85–90% of the data rate/channel capacity of the network; energy consumption in the range of 3.0–3.6 joules, representing 88–95% energy efficiency of the network; effected network range of 2.98, representing 72–89% improved overall performance of the network; and time complexity in the range of 0.20 s, representing 72–89% efficiency of the proposed SDAAA approach. Therefore, our proposed SDAAA protocol outperforms other known approaches, such as SD, EEHA, HAS, IIF, and RHC, designed for secure data aggregation in a similar environment.

## 1. Introduction

Wireless sensor networks (WSNs) can achieve transmission when the network senses data between a node and another set of sensor nodes (SNs). During data transmission in WSNs, sensed data may require significant amounts of energy in terms of power consumption, energy dissipation, etc. [1]. The sensor nodes (SNs) gather the information through event monitoring from their environments and transfer it to the next SN or the base station (BS). However, WSNs obtain data from vulnerable deployment environments through the Internet or sensor actuators. WSNs are usually deployed in several applications such as battlefield surveillance, disaster recovery [2], healthcare applications [3], homeland security [4], agricultural monitoring [5], environmental monitoring, home automation, oil-refinery monitoring process, industry applications, etc. [6,7]. These WSN applications usually occur in vulnerable conditions. Each SN in a WSN is associated with resource constraints such as restricted battery power and limited resources.

Regarding computational capability [8], the sensor nodes deployed in the event-monitoring area often experience redundant data transmission. As a result, the nodes consume additional energy, and congestion may occur in the network when the redundant data are transmitted to the base station (BS) [9]. In addition, different energy consumption may occur due to redundant data transmission. Consequently, aggregating the data locally and adjusting the data to become redundant-free data packets before advancing the data to the BS is urgently desired. After a SN collects identical data packets and makes a single copy of the data, an aggregator node forms. The aggregator node can omit the redundant data using several methods, such as artificial intelligence or probabilistic or statistical approaches [10,11].

Transmitting redundant data is a concern in secure data aggregation. The transmitted data must hop multiple times before the data reaches the BS. Consequently, it becomes inefficient to transmit redundant data in the network. In addition, it is inefficient to hop redundant data because WSNs have limited computational capability and constrained memory of sensor nodes (SNs). The BS/SN discovers such redundant data and drops it. By performing this action, it improves the lifetime of the network, and thus, the energy consumption is reduced, and any delay introduced in the network is also reduced.

Securely aggregating sensed data via aggregator nodes plays a vital role in conserving energy efficiency in the network. In aggregating the sensed data, BS utilizes a security control mechanism. Secure data aggregation (SDA) uses security control mechanisms like authentication and authorization methods in WSNs. Thus, secure data aggregation ensures confidentiality, integrity, and availability for privacy preservation [12] in a WSN while avoiding communication overhead (CO). Thus, secure data aggregation uses sensed data and avoids communication overhead. Consequently, SDA enhances the energy utilization of the SN during sensed data collection. In addition, SDA protocols play a vital role. They act as a firewall that should authenticate and authorize legitimate SN data for privacy preservation against any vulnerabilities or cyberattacks in the network. Due to this limitation of security control mechanisms such as secure node authorization, the lifetime of the WSN becomes a significant challenge. This challenge is due to the need to approve authenticated and authorized legitimate sensor nodes (SN) for secure data transmission in the network, which would subsequently leads to privacy concerns.

Thus, there is an urgent need for a privacy-preserving secure data aggregation approach to handle data aggregation issues and appropriately address data privacy concerns to maintain QoS provisioning [13] in the network. Secure data aggregation is an indispensable paradigm that removes data redundancy and prevents additional energy consumption [14], which requires further security design investigation. Therefore, researchers have introduced privacy-preserving aggregation of time-series data schemes for WSN and uploaded encrypted values for the data aggregation. A data aggregation process calculates the sum of the participants’ SNs. However, it needs access to learn the contents of the data due to limitations in authorization and authentication security control mechanisms in the network, leading to communication overhead (CO). 

However, communication overhead (CO) is a significant concern in WSNs because of the constrained features of sensor nodes. Secure data aggregation and privacy preservation of SN that extend network lifetime reduce CO. Reducing CO leads to sensed data in data aggregation, which helps make essential decisions in the WSN application. Nevertheless, the accuracy of final aggregation outcomes is significant. Therefore, researchers proposed an Energy-efficient and high-accuracy (EEHA) approach for secure data aggregation [15]. EEHA aims to achieve accurate data aggregation without sharing private sensor information and reducing communication overhead. The proposed EEHA achieved its objectives of reducing communication overhead. However, the proposed solution only partially applied to realistic situations from a privacy-preserving secure data aggregation security standpoint. False sub-aggregate values are a security threat for the WSNs created by compromised sensor nodes. In this type of attack, significant errors are generated at the base station. The false sub-aggregate attacks restrict the authentication and authorization process of the nodes, since the EEHA protocol requires authorization of each SN in the network. 

As a result of limited authorization, a false sub-aggregate error synopsis diffusion (SD) [16] approach reduced only false errors in WSNs. SD involves the algorithm that enables the base station (BS) to securely calculate the sum in the presence of those vulnerable or compromised attacks. Furthermore, the SD algorithm helped to calculate the actual aggregate to avoid the contributions of conceded sensor nodes in the aggregation hierarchy. However, cyberattacks like sybil node and sinkhole node [17] cyber threats are prominent vulnerabilities or attacks that compromise legitimate nodes’ confidentiality, integrity, due to limited authentication and authorization. As a result, the sensor node’s transmitted data are not secure.

Accordingly, the authors in [18] proposed a hybrid secure data aggregation (HSA) approach that provides end-to-end secure data communication for WSNs. HSA aims to reduce communication overhead. The approach used a symmetric key-based privacy homomorphism method to guarantee the sensor nodes’ data reading to maintain the network’s privacy. Furthermore, HSA efficiently dealt with key management issues. However, key management issues lead to energy efficiency concerns when maintaining end-to-end data communication. HSA aggregation methods try to use averaging functions to perform the aggregation. However, method like HSA is highly vulnerable to node compromises and all forms of cyberattacks, due to limited secure authentication and authorization.

Therefore, an iterative filtering method was proposed in WSNs to annul these attacks. Iterative filtering algorithms concurrently aggregate data from multiple sources to safeguard the confidentiality level of these sources. However, the iterative filter could not achieve its aim. Subsequently, an improved iterative filtering (IIF) [19] approach successfully handled sophisticated collusion attacks using an initial approximation algorithm in a WSN. IIF focused purely on a single collision attack. However, IIF has limitations in QoS provision, including authentication and authorization security control mechanisms. Therefore, a WSN approach known as a renewable hash chain (RHC) [20] provides confidentiality, authentication, and integrity in WSNs. However, there were no QoS metrics and no authorization security control mechanism in the WSN. 

In this study, we consider secure data aggregation that utilizes both authentication and authorization (SDAAA) to enhance the energy efficiency and privacy preservation of the sensed aggregated data in a WSN. We also identify vulnerabilities and cyberattacks that affect confidentiality, integrity, and availability in SNs, to enhance privacy preservation. Consequently, the proposed SDAAA secure data aggregation protocol in WSN improves QoS performance metrics, such as accuracy in sensed data, that confirms the energy efficiency of the network [21]. Our proposed SDAAA approach maintains the tradeoff between authentication, entity-based authorization, and freshness while maintaining the QoS parameters and energy efficiency. We contribute as follows.

- Our approach provides an entity-based authentication and authorization process that allows only legitimate sensor nodes to communicate and only collects sensed data from those honest sensor nodes in the network. However, unlike WSN privacy-preserving methods such as SD, EEHA, HAS, IIF, and RHC, our approach uses a multi-feature authentication methods.
- In our proposed network architecture, SDAAA utilizes the base station (BS) in the network to authenticate and subsequently authorize the data aggregator node to drop the aggregated packets of those nodes which are not issued a tag to be part of the network. By contrast, SD, EEHA, HAS, IIF, and RHC approaches do not include such security control mechanisms.
- Our proposed SDAAA approach ensures data validity via a novel data freshness mechanism. By contrast, SD, EEHA, HAS, IIF, and RHC do not have any data freshness mechanisms.
- In our proposed SDAAA approach, we determine the sensor nodes’ energy efficiency (EF) before sending the data to that node. In addition, we determine residual energy after the ‘N’ number of communication rounds. In contrast, SD, EEHA, HAS, IIF, and RHC approaches have no EF computation mechanisms.
- Our proposed SDAAA approach detects sybil and sinkhole nodes and other vulnerabilities or cyberattacks when injected into the network. The attacks may be from either malicious outside or inside adversaries. In contrast, SD, EEHA, HAS, IIF, and RHC do not detect all these vulnerabilities or other forms of cyberattacks.
- Our proposed SDAAA approach includes reliability vs. malicious trend of the nodes, throughput performance, and energy efficiency in the presence of malicious nodes. In addition, SDAAA protocol utilizes time complexity, and resilience time of the effected network that provide reliability in the network. In contrast, other protocols like SD, EEHA, HAS, IIF, and RHC do not have such reliability mechanism in their network.

The remainder of the paper is as follows: Section 2 discusses related work, challenges, privacy preservation, and design objectives. Section 3 presents the system model and architecture, attacker model, secure data aggregation using authentication and authorization encryption, and data freshness models. Section 4 is the simulation setup and experimental results. Finally, Section 5 concludes the entire work.

## 2. Related Work and Design Challenges 

### 2.1. Related Work on Privacy Preserving Wireless Sensor Network including Secure Data Aggregation

The authors in [22] proposed preserving data and key privacy (PDKP) data aggregation for wireless sensors that attained data and privacy protection of keys in data aggregation in WSN. Using simple techniques, PDKP uses encrypted data without exposing data content and its key to other SNs. The protocol preserves the key and the data content from an adversary while using less computational overhead. PDKP has no authentication and authorization mechanism in the proposed method.

In [1], the authors proposed an energy-efficient and privacy-preserving data aggregation algorithm (EPDA) for less energy consumption, decreased energy consumption, and prolonged network lifetime. Sensor nodes (SNs) were organized in a tree and connected to the tree’s leaf nodes to form chains. The EPDA used only sensed data through the tail SN nodes of the chains, which were sliced to ensure privacy in the network. However, no authentication and authorization mechanisms are in the proposed method.

The authors in [23] proposed secure data aggregation to preserve data and key privacy (SAPDKP) in wireless sensor networks with multiple sinks to protect keys and data in the data aggregation to preserve data and critical privacy in WSNs with multiple sinks. Multiple sinks consume less energy in computation and communication overhead. However, security concerns include data confidentiality [24], data integrity freshness [25], and data authentication. SAPDKP uses a straightforward technique to perform aggregation and encryption, and uses no authorization security measures. 

In [12], the authors proposed multi-dimensional privacy-preserving average consensus (MPPAC) in wireless sensor networks as a solution to the privacy-preserving average consensus (PPAC) [26] problem only; this solution focused solely on a one-dimensional state, not a realistic simulation of an actual scenario. MPPAC divides nodes into two types: sink nodes and ordinary nodes. MPPAC was used to introduce a super-increasing sequence and an RSA algorithm into the network. This super-increasing sequence played a key role and dealt with any multi-dimensional measurement in the sensors, while the RSA achieved privacy, preserving average consensus only among sink nodes—however, the proposed method needed authentication and authorization mechanisms.

In [27], to create an efficient privacy- and integrity-preserving data aggregation in WSNs, the authors proposed an aggregation scheme for multiple applications (PIMA) in WSNs, using homomorphic encryption which aggregates hybrid sensor data into real WSN applications. The proposed protocol had to satisfy multi-application environment requirements and make use of sensors deployed in heterogeneous environments. PIMA employed Paillier encryption and homomorphic MAC to protect data privacy and check the integrity of the aggregated data. However, there were no authentication and authorization security mechanisms in place. The authors in [28] proposed a design for a privacy and energy-efficient data aggregator (EPSDA) for WSNs. The EPSDA was proposed to overcome energy-intensive aggregator node decryption that reveals a large amount of privacy-protected information to adversaries of the network, which result in the generation of inaccurate results. The proposed EPSDA protocol overcame these limitations and performed direct aggregation on the data, encrypted using homomorphic encryption. The EPSDA restricted old data transferred in the network. However, authentication and authorization mechanisms were not proposed in the scheme.

The authors in [29] proposed a novel energy-efficient and privacy-preserving data aggregation algorithm for WSNs known as CBDA (chain-based data aggregation). CBDA organized sensor nodes (SNs) into tree topology, where leaf nodes sequentially reconnect with each other and form chain topologies. Tail nodes in the topology ensure data privacy by gathering and slicing sensing data into fragments. The CBDA method did not use any authentication and authorization mechanisms in the proposed scheme.

The privacy-preserving secure data aggregation approach involves two main techniques: the first technique applies cryptography to allow the aggregation process to decrypt the data aggregation or sum with different keys. The second technique uses the differential privacy approach to protect data from being compromised [30]. Consequently, the authors in [31] proposed an energy-preserving approach named secure data aggregation watermarking (SDAW) based in homogeneous WSNs. SDAW used lightweight, fragile watermarking without encryption to guarantee integrity and authentication. Malicious nodes in the network can attempt to inject false data to mislead the nodes and gather important information due to the limitation of authorization in the network. The proposed SDAW did not use any authentication and authorization security mechanism in the proposed scheme.

Therefore, adversarial or malicious nodes in WSNs result in confidentiality concerns and compromise data. The compromised data cause additional energy consumption in the network and subsequently lead to communication overhead. As a solution to compromised data, a practical secure data aggregation (SDA) approach was proposed in [32] to ensure data privacy and prevent excess energy consumption. This SDA approach used additive homomorphic, identity-based signatures and batch verification schemes with a proposed algorithm to filter false data injected by malicious nodes without authorization, leading to communication overhead concerns. However, the proposed SDAAA security mechanism methodology needs an authentication and authorization mechanism.

These design challenges in the privacy-preserving secure data aggregation protocols and well-known approaches such as SD, EEHA, HAS, IIF, and RHC (discussed in Section 1) must be outlined and investigated in WSNs.

### 2.2. Design Challenges of SD, EEHA, HAS, IIF, and RHC, including Other Privacy-Preserving Secure Data Aggregation Protocols

As noted in the preceding discussion, privacy-preserving wireless sensor networks’ secure data aggregation protocols and the contending protocols, such as SD, EEHA, HAS IIF, and RHC, experience various network design challenges based on the following.

1. SD, EEHA, HAS, IIF, and RHC secure data aggregation protocols incur QoS concerns and communication overhead due to the absence of authentication, encryption, and authorization [33] security control mechanisms. The absence of these security control mechanisms leads to privacy preservation concerns such as data confidentiality and data integrity.
2. SD, EEHA, HAS, IIF, and RHC secure data aggregation protocols and other privacy-preserving wireless sensor network secure data aggregation incur access control security mechanism concerns. The access control security limitation leads to data redundancy because of limited authorization security control mechanism that would authorize only legitimate SN to be part of the network.
3. SD, EEHA, HAS, IIF, and RHC secure data aggregation protocols, and other privacy-preserving wireless sensor network secure data aggregation protocols encounter network throughput issues. The throughput issue is due to vulnerable SNs and all other cyberattacks. Hence, privacy protections were unavailable in their networks due to limited authentication and authorization security control mechanisms in the networks.
4. SD, EEHA, HAS, IIF, and RHC secure data aggregation protocols and many other privacy-preserving secure data aggregation protocols experience energy efficiency issues, leading to a shorter sensor node lifespan. Therefore, improving a network’s residual energy efficiency algorithm using authentication and authorization security control mechanism is essential to ensure the network’s availability.
5. SD, EEHA, HAS, IIF, and RHC secure data aggregation protocols and many other privacy-preserving wireless sensor network secure data aggregation protocols have experienced the ability to manage node failure. The node failure is due to limitations in secure authentication and authorization in the sensed data, which lead to privacy, reliability, and integrity of data concerns in the network.
6. SD, EEHA, HAS, IIF, and RHC secure data protocols incur scalability regarding large-scale sensor deployment concerns. Scalability of legitimate sensor nodes to be part of the WSN requires availability of well authenticated and authorization security control mechanism.

Therefore, it is important to solve these security challenges in WSN secure data aggregation and privacy-preserving protocols, including well-known WSN approaches such as SD, EEHA, HAS, IIF, and RHC. We first provide an overview of privacy preservation and design objectives, including secure authentication and authorization. After that, we describe the design of a new system model, specifically a mathematical and analytical secure data aggregation model, using our proposed SDAAA protocol methods to assess new QoS performance metrics in the network.

### 2.3. Privacy Preservation and Design Objectives 

In WSN applications like WBANs (wireless body area networks), privacy, secure authentication, and authorization of patients’ information are in high demand. WBANs include emerging widespread wireless sensor network applications, especially in intelligent WSN healthcare applications. Secure authentication and authorization ensure that collected, stored, and transmitted data cannot be accessed and modified by unauthorized agents in networks, such as cyber hackers. Privacy preservation ensures that only authorized agents can access and use data in the network. Consequently, it becomes imperative to address authentication, authorization, and privacy protection concerns for WBANs [34]. 

Cyber hackers could launch various attacks, specifically cyberattacks, including sinkhole node and sibyl node attacks, that could breach healthcare data privacy. Healthcare data privacy is a significant issue leading to the need for protection in wireless sensor networks. Therefore, we focus on the data privacy protection issue resulting from cyberattacks, including sinkhole node and sibyl node attacks that can occur during healthcare WSN patient information monitoring. Cyber threats, including sinkhole node attacks, are internal attacks that compromise network nodes. As a result, compromised or vulnerable sensor nodes (SNs) attempt to attract all network traffic from the neighboring sensor nodes and generate fake routing metrics. Cyber threats resulting from sinkhole node attacks cause routing information issues such as acknowledgment spoofing and selective forwarding attacks. They can also send simulated data to the base station.

Conversely, cyber threats resulting from sybil node attacks sabotage the reputation of hop-to-hop communication systems, generating many fake identities. Sybil node cyber threats happen after sinkhole node attacks, so both attacks rely on each other. We assume hackers understand the deployed security mechanism in the wireless sensor networks. Therefore, hackers may be able to compromise a node by applying a radio communication channel at the medium access control (MC) sublayer. After compromising the node, the attacker launches the sinkhole node cyber threats that release privacy data to the cyber enemies, compromising any individual sensor node privacy. The public key cryptographic authentication technique can secure ad hoc networks. Still, it is not compatible with wireless sensor networks due to the highly resource-constrained attributes of the sensor node. 

The proposed SDAAA protocol guarantees energy efficiency and reliability, and the scalable, secure data communication process in this research model leads to privacy preservation in the network. Consequently, the design objectives of the proposed SDAAA approach are to obtain energy efficiency and fast data aggregation with maximum throughput while maintaining data privacy as a tradeoff. As presented in this research, the proposed SDAAA approach depends on the authentication process that helps maintain node authorization security and ensures privacy in the WBAN healthcare system. Our proposed paradigm considers accuracy as a standard for gauging authentication performance and energy efficiency. 

Therefore, we provide a new system design and mathematical analytical secure data aggregation model to reevaluate new network QoS performance as required.

## 3. System Model and Design Objectives

**Figure 1:**
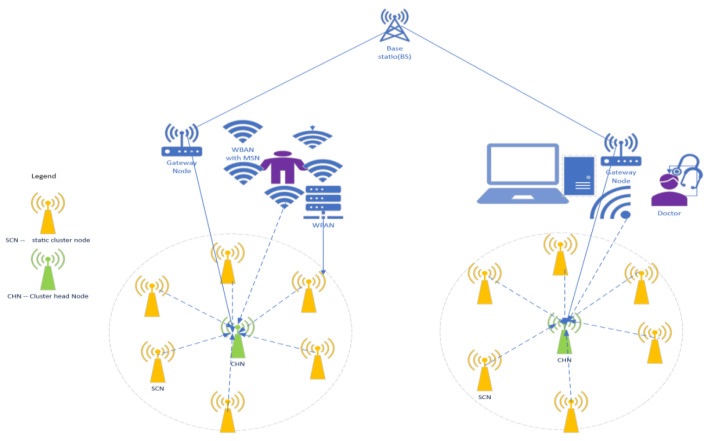
SDAAA healthcare monitoring system architecture model.

To maintain network reliability for QoS performance, confidentiality, integrity, and availability [35] and guarantee privacy preservation [36,37], the proposed SDAAA system model utilizes an intelligent WSN healthcare application monitoring [38] scenario. The WSN application scenario includes a WPAN (wireless personal area network) and a WBAN (wireless body area network) as shown in Figure 1. We monitor patient data and protect healthcare applications (HAs) against all cyberattacks, including sinkhole nodes and sybil node cyber attacks [38,39]. Figure 1 assumes a wireless sensor network consists of a clustered-based topology with limited mobile cluster nodes (MCNs), which are mobile sensors, and static cluster nodes (SCN), which are static sensors, deployed with cluster head nodes (CHNs) in the WSN. However, this cluster provision is unavailable in contending WSN privacy models such as SD, EEHA, HAS, IIF, and RHC protocol system models. Thus, these contending WSN models do not possess MCNs or SCNs or the CHN attributes that enhance network reliability and energy efficiency. However, based upon the cluster network deployment in the proposed SDAAA approach, sensor nodes collectively complete monitoring tasks. 

Due to cost limitations, the sensor nodes are without tamper-resilient hardware. In addition, stationary resourceful heterogeneous sensor nodes are in locations directly connected to the base station (BS). Our proposed SDAAA aggregation system model applies to each cluster in the WSN application, which utilizes four types of sensor nodes in the wireless sensor network: actor nodes (ANs), mobile sensor nodes (MSNs), data aggregator nodes (DANs), and event-monitoring nodes (EMNs). These four types of node provide reliability in the network. By contrast, as stated previously, contending models like SD, EEHA, HAS, IIF, and RHC protocols do not utilize cluster-based network design in their network. Thus, the contending secure data aggregation models do not have the four types of sensor nodes that provide reliability. In our proposed SDAAA approach, the BS is the target point used to compute the aggregation result. The following assumptions are held.

7. Cyberattacks, including sinkhole and sybil node hacks, may occur in healthcare computer systems.
8. EMNs are static cluster nodes (SCNs) that link the static sensor nodes or mobile cluster nodes (MCN) that link the mobile nodes; they are deployed together with the CHNs in the network, and they have the capability to be utilized by WPANs to monitor patients’ health conditions. SCNs or static sensor nodes are deployed in fixed infrastructure equipment. In this research, the EMN communicates with the mobile sensor nodes or MCNs and sensing center to query for patient sensing data.
9. DANs can be either static or mobile sensor nodes that serve as CHNs (cluster head nodes). DANs receive data from the neighboring nodes, aggregate the data, and forward the aggregates to the BS or the gateway. DAN plays a major role in the secure aggregating process. DANs possess more computational and processing capabilities and storage capacity than EMNs.
10. MCNs also link mobile sensor nodes attached to patients and any vehicles within the healthcare environment. They extend the capability of the static nodes in the WSN. MCNs also extend the coverage efficiency of patients and vehicles in the network. Moreover, MCNs can forward updated information to DANs.
11. ANs are actor nodes and can also serve as gateway nodes. They are potent power nodes and processing units for decision processing. ANs have additional computational power and resources responsible for collecting the data from DANs. In addition, they support the BS in identifying legitimate DANs.

**Figure 2:**
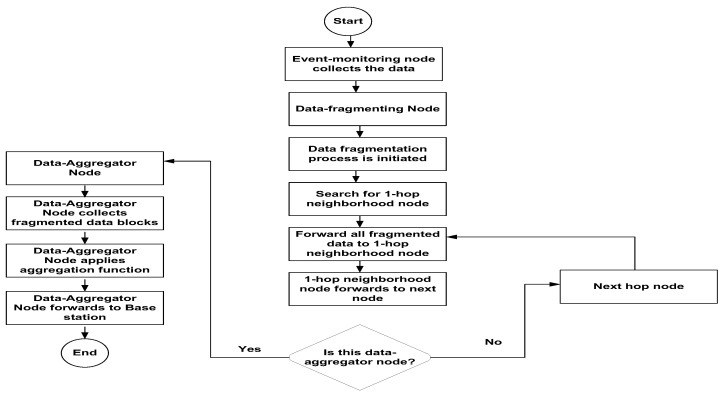
Proposed schematic process for secure data aggregation in WSNs.

Figure 2 initiates a data fragmentation process for secure data aggregation. EMNs are responsible for collecting the data from patient health monitoring events. An EMN forwards the data to the data fragmenting node to fragment data into variable sizes. The data fragmenting node forwards fragmented data to a 1-hop neighborhood, and this fragmenting process keeps moving until it locates a DAN. The DAN collects the data from the data fragmenting node and applies the aggregation processes before sending it to another DAN or the base station.

Furthermore, DANs can communicate with other DANs. A DAN can use typical aggregation functions, including AVERAGE, MIN, MAX, SUM, COUNT, and authentication and authorization features. The additive aggregation functions comprise standard deviation, grouping, and variance that can quickly expand into additive SUM functions [40]. This secure data aggregation supports data fragmentation, as depicted in Figure 1.

### 3.1. Attacker Model

Due to privacy issues such as packet drop and errors of some control packets, signature mismatch may occur. Signature mismatch may lead to the false detection and isolation of inauthentic nodes by the BS (base station). 

Therefore, we use the probability analysis attacker model for detection of any cyberattack such as sinkhole nodes and sybil attacks in the network. Thus, the probability analysis attacker model detects any packet drops or error messages for any malicious node attacks or sensor nodes compromised by cyberattacks. Therefore, the probability analysis attacker model investigates network reliability and reduces any packet drop or errors in the network. This probability analysis attacker model is deployed in our proposed SDAAA protocol. The probability analysis attacker model is not found in any of the contending secure data aggregation protocols. Therefore, we model the packet drop and errors in the network as below.

As proposed in our previous work [41], which is extended by our proposed SDAAA protocol in this research, we optimize the probability of false detection/error by the BS by doubling isolation of any cyberattacks in the network system architecture, which is necessary and is modeled as follows: 

Let

$P_{r}$ = probability of error detection or packet drop in the network

$P_{D}$P = probability of packet drops regarding any privacy concern in the network application

RP = packet generation rate within the network

t = time interval of the BS detecting attack or error in the network

${ASM}_{i}$ = number of authorized signature mismatches of node $N_{i}$ among its neighbors

Then, the probability of ASM exceeding the maximum threshold ${ASM}_{th}$ is as in Equation (1):

(1) $$P_{r}\left(ASM>{ASM}_{th}\right)=1-2\sum \left(\begin{array}{c} RPt\\ i \end{array}\right)P_{D}^{i}{\left(1-P_{Dp}\right)}^{Rt-i}$$

where

*W* = number of warnings received by node *Ni* when any of its neighbors turns out to be malicious or there are vulnerable nodes in the network.

The probability of *W* exceeding the maximum threshold $W_{th}$ is given in Equation (2) as:

(2) $$P_{r}(W\left(N_{j}\right)>W_{th}=1-\sum_{i=1}^{W_{th}}\left(\begin{array}{c} NH\\ i \end{array}\right)P_{FD}{\left(N_{j}\right)}^{i}{\left(1-P_{FD}{(N}_{j})\right)}^{NH-i}$$

where

*NH* is the number of neighbors of node $N_{i}$

$P_{FD}$ ($N_{j}$) is the probability of false detection of node $N_{j}$, which is given in Equation (3):

(3) $$P_{FD}\left(N_{j}\right)=1-exp\left(-2\cdot \frac{{\left(R.t.P_{D}-{ASM}_{th}\right)}^{2}}{R.t.}\right)$$

Then, the probability of doubling detection and Isolating any cyberattacks in the network is $P_{FI}$ and is given by Equation (4) as:

(4) $$P_{FI}\left(N_{j}\right)=1-exp\left(-2\cdot \frac{{\left(NH.P_{FD}\left(N_{j}\right)-W_{th}\right)}^{2}}{NH}\right)$$

### 3.2. Secure Data Aggregation Using Authorization and Authentication in Wireless Sensor Networks (SDAAA)

Secure data aggregation presents the most significant security challenge for privacy in WSNs. The primary objective of secure data aggregation is to enhance the network lifetime. Another objective is to reduce the energy consumption of the sensor nodes through proper use of battery power and efficient bandwidth. However, the data aggregation process may significantly affect QoS, accuracy, and fault tolerance. A decline in QoS and accuracy leads to weak security control mechanisms, such as the limitation of authorizing legitimate sensor nodes (SNs) through authentication and encryption security. Since data aggregation enhances security by reducing redundant data, it could be affected by a compromised or malicious sensor node. Compromised or malicious sensor nodes may illegally obtain the collected data from the neighbor sensor node and report false values as aggregated data. 

In this situation, a malicious node could harm the privacy, confidentiality, and integrity of the confidential data in WSNs. The malicious node could impersonate neighboring or further aggregator nodes. In addition, the attacker may prefer to install a hostile node near the base station to compromise it. As a result, QoS parameters such as energy efficiency, reliability, and accuracy are affected. The fragile design features of WSNs may easily invite destructive cyber attacks, mainly when deployed in unpromising environments.

WSNs comprise resource-restricted sensor nodes with insufficient storage, limited power resources, and lower computational capability. Hence, it is crucial to examine traditional security algorithms in secure data aggregation in WSNs such as SD, EEHA, HAS, IIF, and RHC protocols, as these frailer algorithms cannot meet desired security requirements. Researchers have examined the traditional security balance tradeoffs between security metrics and performance-improving parameters. Secure data aggregation should be reliable, accurate, scalable, and flexible. The reliability, accuracy, and flexibility of the proposed SDAAA approach model enhance fast data aggregation algorithms based on authentication and authorization by the BS (base station). This should be considered when designing secure data aggregation for WSNs. 

Designing protocols meeting single characteristics based on secure data aggregation might be susceptible and exposed to malicious attackers, but multi-featured algorithms can curtail the security risk. Most existing data aggregation methodologies such as SD, EEHA, HAS, IIF, and RHC protocols do not meet these security requirements. Our proposed SDAAA approach utilizes the BS and addresses the node authentication, authorization, freshness, and energy efficiency processes to discourage cyber attacks in a WSN. Furthermore, we focus on improving the QoS provisioning, energy efficiency, and accuracy.

### 3.3. Authorization Process

Prevailing contending proposed secure data approaches such as SD, EEHA, HAS, IIF, and RHC protocols focus only on authentication and ignore the authorization process in wireless sensor networks [42]. Since a newly deployed node could be malicious, a WSN protocol must be cautious when a new node deployment occurs in the network. Newly installed malicious nodes may be difficult to distinguish from legitimate ones due to current wireless sensor network design limitations. A secure data aggregation protocol must prevent hackers from directly deploying malicious nodes. Therefore, it is not enough to protect the wireless sensor networks only from the perspective of node identity; cyberattacks such as sinkhole node and sybil node attacks occur. These attacks introduce malicious nodes that hackers could operate and compromise in the existing legitimate sensor nodes. 

The old deployed genuine nodes possess authenticated and authorized certificates, and the newly created malicious nodes have the same legitimate identities. Therefore, there is a need to distinguish between the old, deployed, and new nodes to defend the network from possible cyber hackers or attacks. The proposed SDAAA protocol utilizes a timestamp technique in both the authentication and authorization processes. It maintains freshness to avoid the potential threats and hacking of the data; this feature is unavailable in approaches such as the SD, EEHA, HAS, IIF, and RHC protocols. Thus, our secure authentication process guarantees authentication, authorization, and freshness. In our proposed SDAAA approach, Algorithm 1 describes the authorization procedure as follows:
**Algorithm 1.** Authorization process for legitimate and non-legitimate sensor nodes**1.  ** Initialization: ($K_{auth}$: authorized Sensor node; Auth: authorization; k: sensor node; $k_{leg}$: legitimate sensor node Me: Message; ${BS}_{n}$: Base station; $C_{auth}$: Certificate for authorization; $E_{BS}$: expected action performed by base station $W_{sn}$: Wireless Sensor Network; $k_{id}$: Sensor node’s identity)**2.  ** Input: ($Me,C_{t},k_{id},{PU}_{K},C_{e}$)**3.  ** Output: {(Auth),$C_{auth}$}**4.  ** Set k = $W_{sn}$**5.  ** If $W_{sn}$ allows entry to the ‘sensor node, then.**6.  ** ${BS}_{n}$ Starts $E_{BS}$ for k**7.  ** ${BS}_{n}$ releases $C_{auth}$ for $k_{leg}$: $C_{auth}C_{auth}=h\left\{k_{id},C_{e}∥{PU}_{K}\right\}$** // released certificate for legitimate node.****8.  ** Set Authfork //$Auth=\{Me,t_{c},{Sig(BS}_{n})\left\{\left(k_{id}∥t_{c}∥Me\right)\right\},C_{k_{id}}$**// Authorization process.****9.  ** end if**10.** ${BS}_{n}$ broadcasts Auth (Me) for the k node in $W_{sn}$**11.** $If\;k\in K_{auth}$, then**12.** $K_{auth}$ reads Auth (Me)**13.** Set Auth = ***k*** node ∈ $K_{auth}$**14.** else if Auth≠k**15.** ${BS}_{n}declinesaccesstheKnode$**16.** end if**17.** end if else

Algorithm 1 shows the authorization process. In lines 1–3, we describe the parameter-initialization, input, and output processes, respectively. In line 4, the sensor node forms part of the network to participate in the data-monitoring/data-gathering process. In lines 5–7, if the WSN permits the sensor node to join the network, it performs a particular action as a guaranteed authorization certificate. As a result of the authorization certificate issued by the BS, the BS authorizes the legitimate sensor nodes to send and receive data in the network in line 9. In lines 10–13, the base station (BS) only broadcasts the message to authorized nodes to continue communication with them. Thus, only nodes authorized by the BS can read the messages in the network. In lines 14–15, the node is checked; if the node does not have authorization, then data communication with that node is stopped. Table 1 shows the variables used in the broadcasting message and certificate.

### 3.4. Authentication Formation

Here, we show the performance of the entity-based authentication features of the proposed SDAAA model that helps the base station discover any falsely aggregated data. This is unlike the contending WSN approaches such as SD, EEHA, HAS, IIF, and RHC protocols, which do not utilize entity-based authentication in their network. Let us assume that sensor node ‘*k*’ is a compromised node and launches false sub-aggregate data by inserting a few wrong data bits into the collected aggregated data. To prevent this, the base station broadcasts the data aggregation query message that uses the random value ‘$R_{val}$’. In response, sensor node’ km sends its message authentication code (*MAC*) to the base station to authenticate the sensed value ‘$S_{v}$’. The node ‘*k*’ uses ‘$R_{val}$’ and its own identity ‘$N_{id}$’ to compute the *MAC* as follows:

(5) $$N_{MAC}=\sum k\left(R_{val}\right)+N_{id}$$

Based on the computed *MAC* address, the base station generates the random value and can also determine the falsely inserted portions of the aggregated data:

(6) $${BS}_{n}=G+\sum {{(R}_{val})}^{\beta }\times k_{n}$$

where *G* is the random generator, β is the number of generated values based on the number of sensor nodes, and $k_{n}$ is the total number of sensor nodes in the network.

Then, the base station ‘${BS}_{n}$’ generates the random value that determines the inserted false data bits in aggregated data given by:

(7) $$B_{f}=\sum \left({BS}_{n}\right)\times N_{MAC}≃D_{a}$$

where $D_{a}$: aggregated data, $N_{MAC}$: number of MAC addresses, and $B_{f}$: false data bits that are available in aggregated data.

**Lemma 1:**  *The hacker cannot generate an MAC associated with the false data aggregation data bits* ‘$B_{f}$’ *that the base station cannot discover as false*.

**Proof:**  Let us assume if sensor node ‘k’ contributes the dat bits $B=\left\{b_{1},b_{2},b_{3},\ldots ,b_{n}\right\}$ in its local summation ‘γ∇’, then it generates the MAC for authentication purpose ‘$k_{MAC}$’ written as $k_{MAC}\left(A_{k},K_{l}\right)$.

Where $A_{k}$: secret key generated by sensor node ‘k’ and ‘$K_{l}$’: key length.

The sensor node ‘k’ shares this key with base station ‘${BS}_{n}$’ with length ‘$K_{l}$’ Thus, the characteristics of the key ‘$K_{c}$’ can be written as:

(8) $$K_{c}={[K}_{e}(B+R_{v})]{\to BS}_{n}$$

where $K_{e}$: the encrypted key, which encrypts the data bits before sending them to the base station.

Each legitimate node ‘$L_{n}$’ in the network appends the key length ‘$K_{l}$’ with ${(K}_{l}+K_{l}^{*})$ which is the same as:

$${(K}_{l}+K_{l}^{*})={[K}_{e}(B+R_{v})]$$

Thus, the key can be simplified as follows:

$${{(K}_{l}+K_{l}^{*})=K}_{c}$$

$$K_{Ex}={(K}_{l}+K_{l}^{*})$$

(9) $$K_{c}=K_{Ex}$$

where $K_{Ex}$: extended key length. □

Let us assume the sensor node’s MAC address ‘$k_{MAC}$’ is compromised and the extended key ‘$K_{Ex}$’ reaches the base station ‘${BS}_{n}$’. Another sensor node cannot inject the key of node ‘k’ without detection. We observe that ‘$K_{Ex}$’ cannot include false data bits of aggregated data. This feature helps maintain the authentication process in the SDAAA approach.

#### 3.4.1. Authentication and Encryption

Secure data packet transmission in our proposed SDAAA approach can increase authentication and authorization utilizing encryption. Furthermore, it maintains the freshness of the message, which limits hackers’ access to data. Contending approaches, such as SD, EEHA, HAS, IIF, and RHC protocols, only use authentication without any authorization or encryption process. Let us assume that the sensor node ‘km’ forwards the data to the cluster head ‘$C_{H}$’. The encrypted packet, data sent to the cluster head, and data payload are as shown below:

(10) $$\left[P_{E}=\left\{k_{id},\;C_{H_{id}},\;R_{v},\;M_{k\;}\left(k,\;CH\right).P_{data}\times \left(∆d\right)\;\right\}\right]\;$$

In Equation (10), the packet encrypted procedure includes sensor node ‘*k*’ and its identity ‘$k_{id}$’, cluster head ‘*CH*’ and its identity ‘C’, randomly chosen value for encrypting the data $R_{v}$ MAC’s key ‘$M_{k}$’. In addition, data payload ‘$P_{data}$’ and the amount of data sent ‘∆d’ occur. The base station and other sensor nodes can identify the packet’s source based on the inserted information.

(11) $$∆d=k_{id}∥C_{H_{id}}∥R_{v}∥P_{data}$$

Equation (11) shows the amount of data sent in different forms from different sources (e.g., node’s identity, cluster head’s identity, randomly generated value, and data payload).

(12) $$P_{data}=M_{k}\left(k,CH\right)k_{r}$$

Equation (12) shows the data payload format, and it involves the MAC key ‘$M_{k}$’ generated for the sensor and cluster head node and the sensor node’s reading ‘$k_{r}$’ obtained through an event-monitoring process.

The data sent using the MAC key ‘$M_{k}$’ complies with the message authentication code of the data. The cluster head node ‘$C_{H}$’ receives the aggregated encrypted packet ‘$A_{EP}$’ from node ‘k’ and forwards it to the next hop node ‘$N_{C_{H}}$’, which could be either another sensor node ‘$k_{a}$’, cluster head node ‘*CH*’ or base station ‘${BS}_{n}$’. Thus, Equation (13) shows the aggregated encrypted packet as:

(13) $$A_{EP}=\left[k_{a}∥C_{H_{id}}∥{BS}_{n}\left(id\right)\{P_{data}\;\}\times R_{v}\right]$$

where ${BS}_{n}\left(id\right)$: base station’ identity.

Equation (13) shows secure data sent via encryption $e\left({∆d}^{\prime }\right)$ after applying the aggregation process ‘$D_{ag}$’.

(14) $${e({∆d}^{\prime })(}_{Dag})=\left({GK}_{ran}\left\{(CH∥{BS}_{n}).∆d1\right\}+k_{a}∥CH(∆d2)\right)$$

where ∆d1 denotes data shared between the cluster head node and the base station; ∆*d*2 denotes data transmitted between the next-hop sensor node and the cluster head node.

We observed that for data ${e({∆d}^{\prime })(D}_{ag})$, the attacker or hacker could not modify data e(∆*d*^′^)(*D_ag_*) because they were fully encrypted using the group’s secret random keys ‘${GK}_{ran}$’. 

Where ${GK}_{ran}$: group of secret random keys shared by either two cluster head nodes or cluster head nodes and base station.

Equation (14) shows fully encrypted shared data ${∆d1}^{\prime }$ between cluster head node ‘*CH*’ and base ‘${BS}_{n}$’ station as:

(15) $${{∆d1}^{\prime }=e\{BS}_{n}\left(id\right)+C_{H_{id}}\}+R_{v}{\times P}_{data}$$

where ∆d2 denotes data transmitted between the next hop sensor node and cluster head.

In Equation (16), the secure data transmitted between the next-hop sensor node and cluster head ${∆d2}^{\prime }$ is given by:

(16) $${∆d2}^{\prime }=\left\{e\left(k_{a\left(id\right)}∥C_{H_{id}}\right).∆d2+(R_{v}{\times P}_{data})\right\}$$

$k_{a\left(id\right)}$: next hop sensor node’s ID; e: identity encryption process

By combining the features of equations from above, we obtain the secure data aggregation and authentication processes, which leads to QoS ‘$Q_{pr}$’ in the WSN as follows:

(17) $$Q_{pr}=A_{EP}+{∆d1}^{\prime }+{∆d2}^{\prime }+{e({∆d}^{\prime })(D}_{ag})$$

#### 3.4.2. Secure Data Freshness Process

We observed the sensor reading ‘$k_{r}$’ obtained by the sensor node ‘*k*’, and resultant aggregation listening capability ‘ε’ of the cluster head node ‘$C_{H}$’ in the premises of any number of events $E_{n}^{\prime }$. This is important for securing the freshness of the data. Thus, every valid sensor node ‘k’ belongs to its respective ‘$C_{H}$’ and has a total number of sensor nodes ‘$k_{n}$’, which can forward the valid ‘$V_{u}$’ and invalid ‘$I_{u}$’ updated information to the cluster head $C_{H}.$ Hence, the current detection time ‘$D_{t}$’ for the right communicating cluster head node ‘$V_{CH}$’, conforms to the proposed SDAAA approach; however, it is absent in the other contending approaches like SD, EEHA, HAS, IIF, and RHC protocols. The proposed SDAAA approach secure data freshness is as shown below:

(18) $$D_{t}=\frac{\left(k_{r}*E_{n}\right)\times k_{n}}{\varepsilon }$$

Nevertheless, it is a tricky process to identify a valid cluster head node. We assume the right cluster head node ‘$V_{CH}$’ and non-valid cluster head node ‘${NV}_{CH}$’ are available in the network. However, finding the valid cluster head node increases the necessity of secure freshness to avoid compromising the aggregated data. Thus, the right cluster head node ‘$V_{CH}$’ can be obtained by:

(19) $$V_{CH}=\left(\begin{array}{c} V_{CH}+1,D_{t}>\frac{V_{CH}-1}{V_{CH}}\\ V_{CH},otherwise \end{array}\right)$$

Once the valid cluster head node is determined, it is also appropriate to isolate the non-valid cluster head node ‘${NV}_{CH}$’ from the network; otherwise, it could damage the data integrity, which is vulnerable when securing data freshness. Thus, non-valid cluster head node ‘${NV}_{CH}$’ is obtained by:

(20) $${NV}_{CH}=\left(\begin{array}{c} {NV}_{CH}+\tau \left({∆d}_{r}\right),D_{t}>\frac{V_{CH}-1}{V_{CH}}\\ {NV}_{CH},otherwise \end{array}\right)$$

where τ: data-effecting process; ${∆d}_{r}$: resultant aggregated data of cluster head node.

Henceforth, the same process also correctly determines the valid (legitimate) and non-valid (malicious) sensor nodes when forwarding the data in the network.

#### 3.4.3. Energy Efficiency

Communication overhead (CO) leads to additional energy consumption. Therefore, we attempted to reduce communication overhead that occurs in the contending WSN approaches, such as SD, EEHA, HAS, IIF, and RHC protocols as compared to the proposed SDAAA approach, which resolves CO as follows: 

In the proposed SDAAA protocol, let us assume the sensor network ‘$N_{s}$’ consists of the sensor nodes ‘$k_{n}$’, which exchange the messages ‘$M_{1}$’ and ‘$M_{2}$’. Determining the message exchange process between intermediate nodes is of paramount significance for determining the consumed energy of the nodes. Thus, we determine the communication overhead ‘$C_{o1}$’ of the source node as:

(21) $$C_{o1}=\left(1-M_{1}\right)*k_{n}*M_{2}$$

When forwarding data to either the next hop or the destination hops, data dependency ‘$D_{d}$’ may occur at each hop, increasing communication overhead. Thus, the communication overhead for the next hop nodes or destination node, except the source node, is given by:

(22) $$C_{o2}=\left(1-M_{1}\right)*k_{n}\times \sum k*\left(D_{d}-1\right)$$

Equations (21) and (22) show the communication overhead of the messages exchanged between source and destination nodes. Therefore, the total communication overhead ‘$C_{to}$’ of the message exchange can be determined as follows:

(23) $$C_{to}=\left(1-M_{1}\right)*k_{n}+\left(1-M_{1}\right)*k_{n}\times \sum k*\left(D_{d}-1\right)$$

Equation (23) shows that when the overhead ratio ‘$R_{o}$’ of our proposed SDAAA approach is compared with other known approaches, our proposed SDAAA approach communication overhead is much lower, as determined by Equation (24):

(24) $$R_{o}=\frac{\left(\left(1-M_{1}\right)*k_{n}+\left(1-M_{1}\right)*k_{n}\right)+\sum k*\left(D_{d}-1\right)}{M_{1}*M_{2}}$$

Thus, the communication overhead of our proposed SDAAA approach is less than that of the other contending approaches, which helps preserve energy consumption.

(25) $$R_{o}=\frac{\left(\left(1-M_{1}\right)*k_{n}+\left(1-M_{1}\right)*k_{n}\right)+\sum k*\left(D_{d}-1\right)}{M_{1}*M_{2}}<1$$

To determine the energy consumption, the sensing energy ‘$E_{S}$’ of the sensor node ‘*k*’ can be computed as:

(26) $$E_{s}=ή\times R_{s}$$

where ή: a constant representing the energy to sense a bit of data and $R_{s}$: sensing rate in bit/s.

For communication purposes, we calculate the energy dissipated ‘$E_{d}$’ by sensor node ‘k’ to transmit the data packet of size ‘$P_{s}$’ to destination sensor node ‘$k_{d}$’s as:

(27) $$E_{d}=\left(k,k_{a},r,P_{s}\right)=\left((\tau_{1}+\tau_{2}\times r{\left(k,k_{d}\right)}^{nh})\times {(R}_{s}\times P_{s})+R_{o}\right)$$

After the energy dissipation for transmitting the packet is obtained, the energy for receiving the packets ‘$E_{r}$’ can be obtained as:

(28) $$E_{r}=\left(k,k_{a},P_{s}\right)=\left(\left(\tau_{4}*R_{s}\times P_{s}\right)+R_{o}\right)$$

Finally, we determine the total energy consumption ‘$E_{t}$’ for the entire communication process as follows:

(29) $$E_{t}=\left(\left(ή\times R_{s}\right)+\left(\left(\tau_{4}*R_{s}\times P_{s}\right)+R_{o}\right)+((\tau_{1}+\tau_{2}\times r{\left(k,k_{d}\right)}^{nh})\times {(R}_{s}\times P_{s})+R_{o})\right)$$

By substituting the values, we obtain:

(30) $$E_{t}=\left(E_{s}+E_{d}+E_{r}\right)$$

where r: 1hop distance between two sensor nodes set to 1, $\tau_{2},\tau_{3},\tau_{4}$: constant parameters, nh: Number of hops between source and destination nodes. 

## 4. Simulation Setup and Experimental Results 

This research was implemented and secure data aggregation simulated using an authentication and authorization approach for wireless sensor networks. Secure data aggregation involves challenges regarding security, QoS, energy efficiency, throughput, performance, and large-scale network deployment. We generated eight simulation scenarios close to realistic scenarios covering an entire intelligent healthcare WSN application scenario. We tested the generated scenarios and validated them using our proposed SDAAA approach. Our approach was programmed using C++ and was run on an OMNET++ simulator to achieve these goals. We tested each scenario several times to determine the strengths and limitations of our proposed SDAAA approach. We also compared our proposed SDAAA approach with contending approaches such as SD, EEHA, HAS, IIF, and RHC protocols. Finally, we collected the results based on ten simulation runs for each scenario as below.

- Scenario 1: Monitoring the internal data regarding the patient’s condition using a WSN monitoring application to check for inside malicious involvement in the network using static nodes.
- Scenario 2: Monitoring entities outside of the intelligent WSN (e.g., vehicles, deployed persons, etc.) using static and mobile sensor nodes.
- Scenario 3: Monitoring internal and external activities using static sensor nodes.
- Scenario 4: Monitoring internal and external activities using static and mobile sensor nodes.
- Scenario 5: Generating 2% malicious sensor nodes in Scenario 1.
- Scenario 6: Generating 2%, 5%, and 10% malicious nodes in Scenario 2.
- Scenario 7: Generating 5% and 10% malicious nodes in Scenario 3.
- Scenario 8: Generating 5% and 10% malicious nodes in Scenario 4.

The simulation aims to validate the performance of the proposed SDAAA approach in the presence of malicious nodes (internal and external) and detect the impact using QoS metrics. Furthermore, we compared our proposed SDAAA approach with the contending approaches: SD, EEHA, HAS, IIF, and RHC. We used similar parameters for all the methods in the simulation. For simulation purposes, we used a 1300 × 1300 network topology and involved 540 sensor nodes with a transmission range of 40 m. We set the initial energy of the nodes to 5 joules. The node bandwidth was 50 kb/s, and the maximum energy consumption of the sensor nodes for receiving and transmitting the data was set to 13.5 mW and 15.0 mW, respectively. Sensing and idle modes were set to 10.4 mW and 0.45 mW, respectively. The total simulation time/round was 36 min, and the pause time was 15 s, set for phase initialization before starting the simulation. We show the simulation parameters in Table 2.

Based on the simulation, we obtained the following results:

- Average throughput
- Average energy consumption
- effected network vs. resilience time.
- Complexity

### 4.1. Throughput

**Figure 3:**
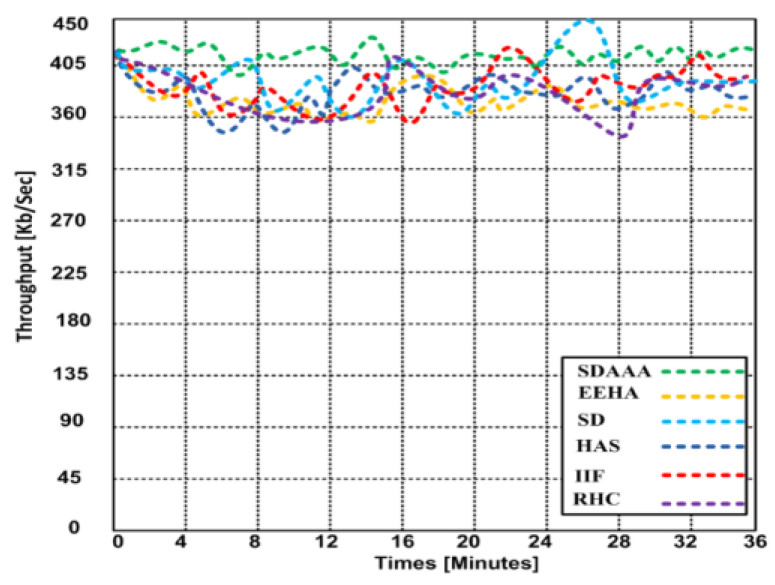
Average throughput of our proposed SDAAA and other contending approaches: EEHA, SD, HAS, IIF, and RHC over entire simulation time.

Throughput is a significant metric generally expressed as the data transmitted over the sensor network. We consider that the source nodes generate a random number of data packets in ranges that can lead to delay/latency and can affect the throughput of the network. We developed several simulation scenarios to measure our proposed SDAAA approach’s maximum throughput and compared it with contending approaches such as SD, EEHA, HAS, IIF, and RHC. Based on the simulation results, we observed that when time increases, the throughput performance of all approaches remains variable; see graphs in Figure 3.

**Figure 4:**
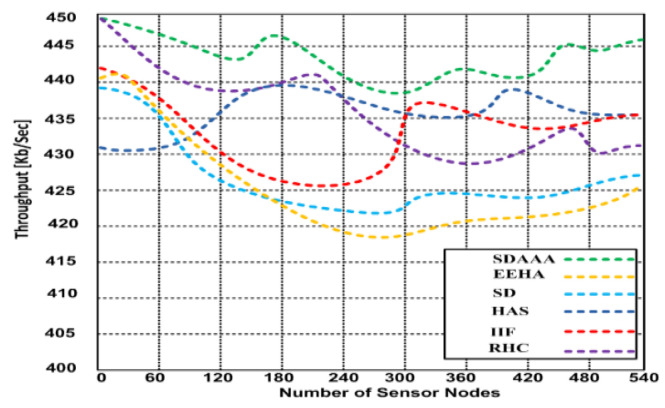
Average throughput of our proposed SDA and other contending approaches: EEHA, SD, HAS, IIF, and RHC in the presence of malicious nodes.

**Figure 5:**
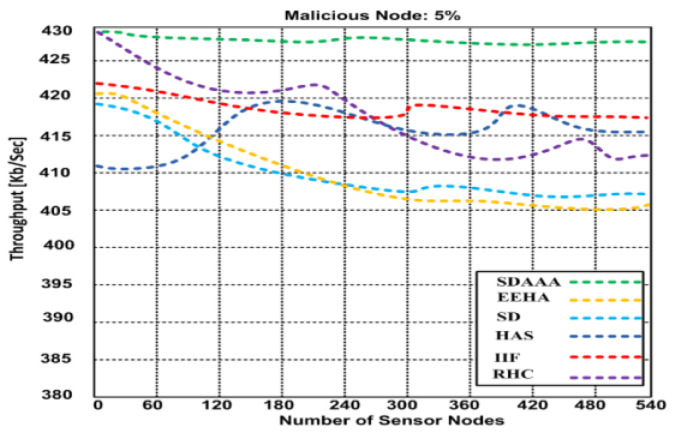
Average throughput of our proposed SDA and other contending approaches: EEHA, SD, HAS, IIF, and RHC in the presence of malicious nodes.

**Figure 6:**
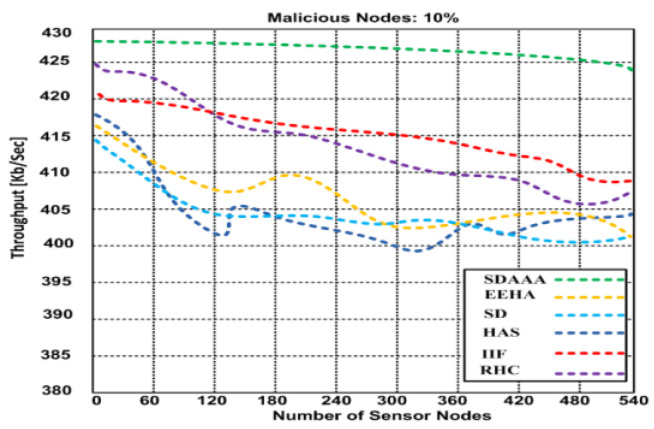
Showing average throughput of our proposed SDA and other contending approaches: EEHA, SD, HAS, IIF, and RHC in the presence of malicious nodes.

However, the throughput performance of our proposed approach was better than that of the contending approaches. Based on the graphs in Figure 4, Figure 5 and Figure 6, our proposed SDAAA approach’s throughput performance is much better than other competing approaches like SD, EEHA, HAS, IIF, and RHC. In Figure 3, the approximate throughput performance of our proposed SDAAA approach is 422 kb/s, while other competing approaches have 361–406 kb/s. 

In Figure 4, the average throughput performance of our proposed SDAAA approach was 444 kb/s. In contrast, the performance of competing approaches like SD, EEHA, HAS, IIF, and RHC protocols was 415–443 kb/s with several increased sensor nodes. As depicted in the graph in Figure 5, we generated 5% malicious nodes to confirm the effectiveness of our approach and other competing approaches. The results show that our proposed SDAAA approach had a 426.5 kb/s throughput performance, which indicates less latency, compared with the competing approaches like SD, EEHA, HAS, IIF, and RHC, which have 405–418 kb/s, indicating high latency. We generated 10% malicious data, as shown in Figure 6. The number of malicious nodes increased, affecting the throughput performance of competing approaches. However, our proposed SDAAA approach is promising. Our proposed SDAAA approach reached 424.5, while other contending approaches like SD, EEHA, HAS, IIF, and RHC reached 402–409 kb/s. The reason for better throughput in our method is the lightweight authentication and authorization process that handles the malicious node’s activities effectively. 

### 4.2. Average Energy Consumption 

**Figure 7:**
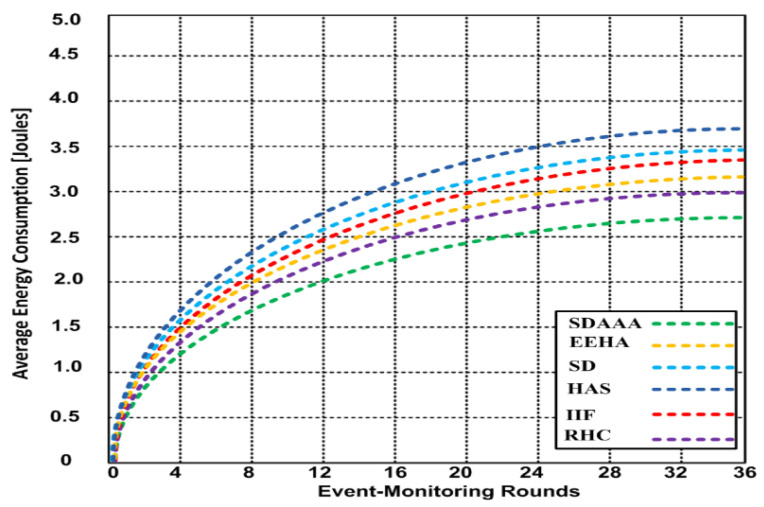
Energy consumption of proposed SDAAA and other contending approaches: EEHA, SD, HAS, IIF, and RHC during different event-monitoring nodes.

Secure data aggregation should focus on improving QoS, so for the network to consume minimum energy for enhanced data communication is paramount. After completing the experiments on the network, we determined the energy salvation process by monitoring and data-forwarding packets to the data aggregation and sink nodes. We generated three scenarios involving malicious and non-malicious nodes. Based on the simulation results, we perceived that the energy consumption upsurges when event-monitoring rounds increase. Figure 7 depicts the result for the non-malicious scenario; 2.6 joules of energy are consumed during the 36 event-monitoring rounds using our proposed SDAAA approach, whereas other contending approaches such as SD, EEHA, HAS, IIF, and RHC consumed 3–3.62 joules. 

**Figure 8:**
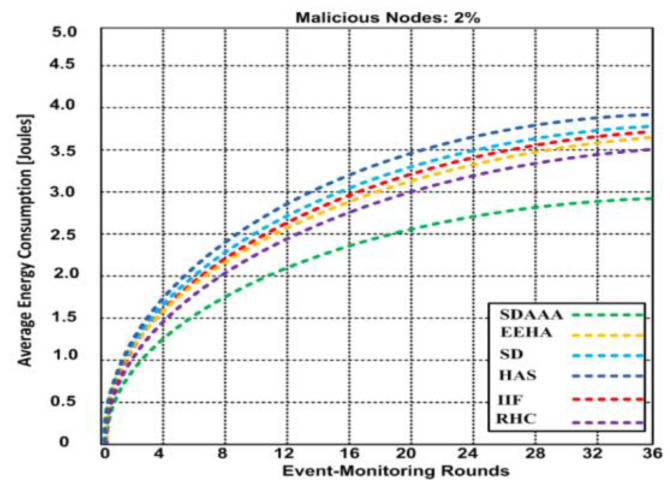
Energy consumption of proposed SDA and other contending approaches (EEHA, SD, HAS, IIF, and RHC) by different event-monitoring nodes in the presence of malicious node.

**Figure 9:**
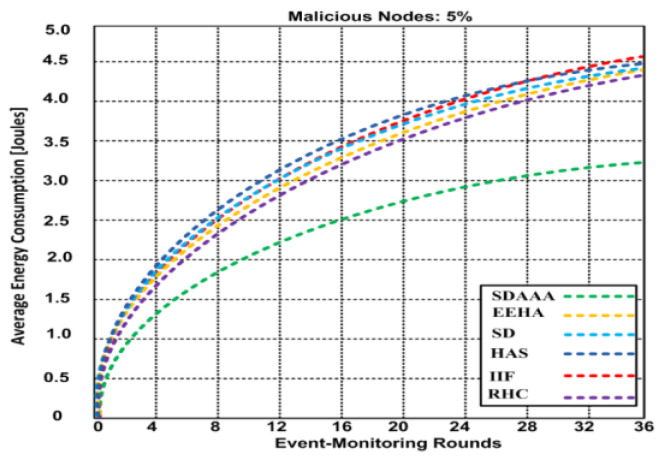
Energy consumption of proposed SDA and other contending approaches (EEHA, SD, HAS, IIF, and RHC) by different event-monitoring nodes in the presence of malicious node.

We generated 2% malicious nodes in Figure 8. We observed that nodes consume more energy in adversarial node scenarios. Our proposed SDAAA approach consumes 2.91 joules over 36 event-monitoring rounds compared to other competing approaches like SD, EEHA, HAS, IIF, and RHC, which consumed 3.5–3.94 joules in a similar scenario. In Figure 9, the generated scenario comprises 5% malicious nodes, and the results show that as the number of malicious nodes increases, additional energy is consumed. The increased number of malicious nodes significantly affects QoS and reduces the network lifetime. Our proposed SDAAA approach required 3.32 joules, while other contending approaches like SD, EEHA, HAS, IIF, and RHC consumed 4.39–4.52% over 36 event-monitoring rounds. Our proposed SDAAA approach consumed less energy overall. The reason behind the minimal energy consumption in our proposed SDAAA approach is the incorporation of an energy-efficient model that helps cause less energy consumption in the network. 

### 4.3. Affected Network vs. Resilience Time

**Figure 10:**
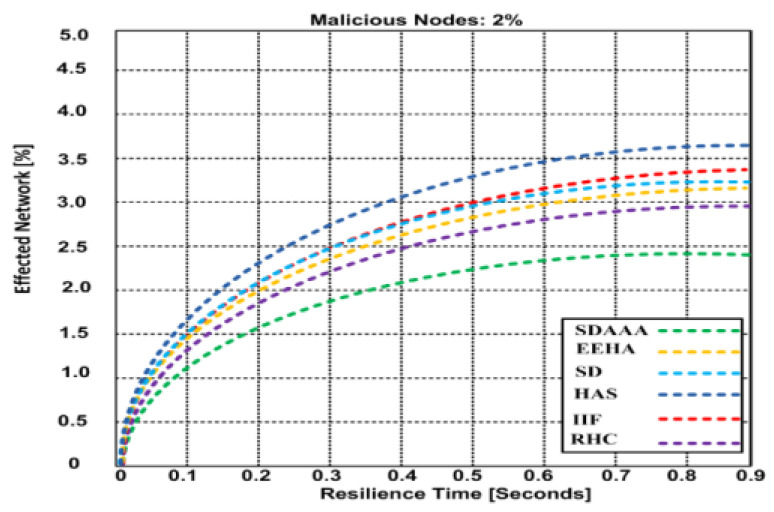
Effected network vs. resilience time of the proposed SDA and other contending approaches (EEHA, SD, HAS, IIF, and RHC) in the presence of malicious nodes.

Regarding resilience time, we determined the performance of our proposed SDAAA approach and other contending approaches such as SD, EEHA, HAS, IIF, and RHC. We generated three malicious scenarios and diagnosed the affected network. We noticed in the results, as depicted in Figure 10, that when a network is affected by malicious nodes, then the resilience time also increases. However, our proposed SDAAA approach obtains minimum resilience time compared to the other contending secure data aggregation approaches like SD, EEHA, HAS, IIF, and RHC. Our proposed SDAAA approach yielded a 2.45% effected network with 0.9 maximum resilience time, whereas competing approaches like SD, EEHA, HAS, IIF, and RHC produced a 2.98–3.62% affected network with 0.9 full resilience time.

**Figure 11:**
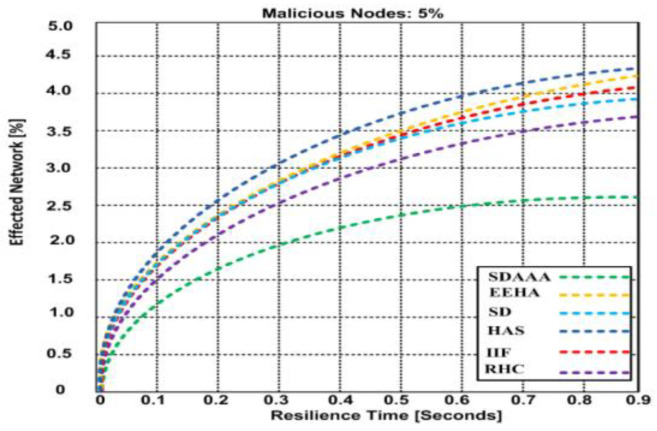
Effected network vs. resilience time of proposed SDA and other contending approaches (EEHA, SD, HAS, IIF, and RHC) in the presence of malicious nodes.

**Figure 12:**
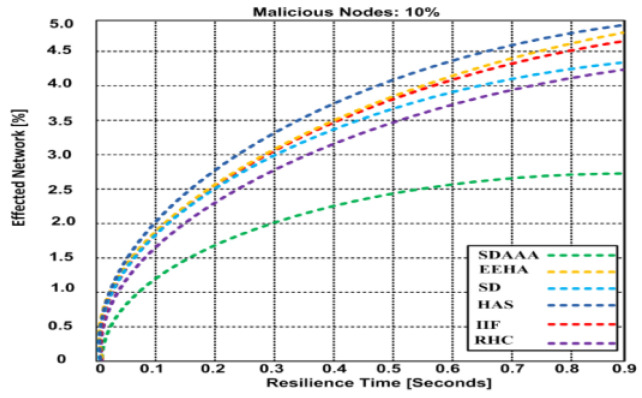
Effected network vs. resilience time of proposed SDA and other contending approaches (EEHA, SD, HAS, IIF, and RHC) in the presence of malicious nodes.

Figure 11 depicts a malicious scenario involving 5% malicious nodes. Based on the results, our proposed SDAAA approach has an affected network of 2.62%, with 0.9% resilience time, whereas other competing approaches like SD, EEHA, HAS, IIF, and RHC have effected network of 3.68–4.38%, with 0.9% resilience time. In Figure 12, the ratio of malicious nodes’ is increased up to 10%. This increment highly affects other contending approaches such as SD, EEHA, HAS, IIF, and RHC, which lead to a high network effect of 4.47–4.99%, while our proposed approach SDAAA is not significantly effected has only 2.67%. We surmise that the better performance of our proposed SDAAA protocol is due to the use of authentication, authorization, and a data-freshness paradigm, which helps the data aggregation nodes to identify false aggregated data accurately. Our SDAAA approach was unaffected, while the other contending secure data aggregation approaches did not provide authorization, authentication, or energy efficiency.

### 4.4. Time Complexity

**Figure 13:**
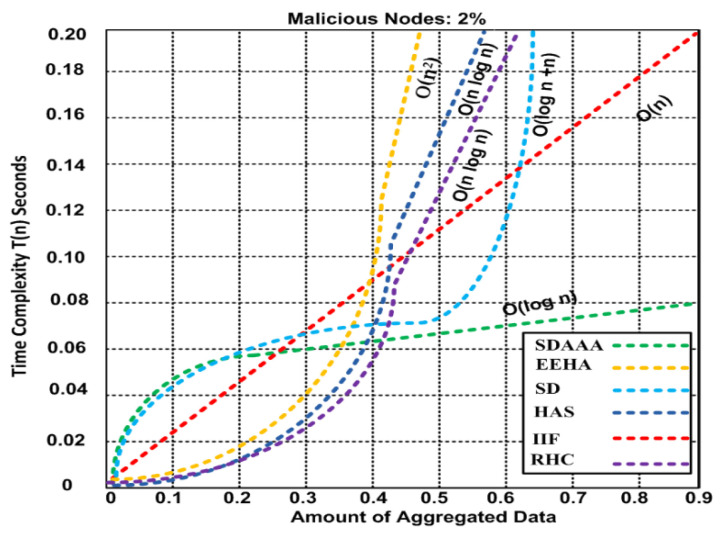
Time complexity of SDAAA, SD, EEHA, HAS, IIF, and RHC.

The time-related performance of any protocol depends on the task’s complexity. Time complexity refers to the time required to run a task, signifying effectiveness in the input. We measure the time complexity by calculating the number of basic operations accomplished by the algorithm. Based upon this, an essential operation takes a constant amount of time to execute. In Figure 13, we draw the trend of the time complexity for our proposed SDAAA approach and compared its time complexity with other contending approaches such as SD, EEHA, HAS, IIF, and RHC. The results confirm that the proposed SDAAA approach had O (log n) time complexity and required 0.08 s to complete 0.9 aggregated data. Time complexity was determined using 2% malicious nodes in the network. 

Our proposed SDAAA approach had the lowest time complexity because it uses secure authentication, authorization, and energy efficiency that help lessen the time complexity. We establish our proposed SDAAA approach and contending approaches on a recursive basis. Therefore, time complexity can be determined using recursive features given by the following formula, and the details are in Table 3.

(31) $$T\left(n\right)=\left(\begin{array}{c} O\left(1\right)\;If\;n=1\\ at\left(\frac{n}{b}\right)+O\left(n\right)\;If\;n>1 \end{array}\right)$$

Based on the time complexity results, we evaluated our proposed SDAAA and the other contending approaches like SD, EEHA, HAS, IIF, and RHC; details are given in Table 4.

The simulation results confirm that our proposed SDAAA approach performs better than the known secure data aggregation approaches like SD, EEHA, HAS, IIF, and RHC. We show the detailed outcomes of metrics used in our proposed SADAA approach, compared with the other secure data approaches like SD, EEHA, HAS, IIF, and RHC in Table 5 as follows:

## 5. Conclusions 

This paper proposes a method of secure data aggregation using the authentication and authorization (SDAAA) protocol for detecting cyberattacks, including sybil node and sinkhole node attacks or hackers in wireless sensor networks. The proposed paradigm aims to monitor and protect intelligent healthcare application systems and personnel from internal and external cyber security threats that can disrupt the functional process of smart healthcare application monitoring. The paradigm uses a node authorization algorithm. The algorithm prevents the entry of prohibited or malicious nodes known as sybil node and sinkhole attacks in the network. Furthermore, the paradigm focuses mainly on authentication, authorization, and freshness, which combat malicious node effects in the network and improve the energy consumption of the WSN. These security mechanisms sustain the tradeoff between energy efficiency, accuracy, and QoS provision in the WSN. 

We programmed in C++ and implemented on the OMNET++ simulator to confirm the legitimacy and strength of our proposed SDAAA protocol. Based on the extensive experimental results, we have validated that our proposed SDAAA protocol has an accuracy of 444 kb/s representing 98% of data rate/channel capacity of the network, an energy consumption of 2.6 joules representing 99% energy efficiency of the network, an affected network of 2.45 representing 99.5% achieved overall performance of the network, and a time complexity of 0.08 s representing 98.5% efficiency of the proposed SDAAA approach. By contrast, contending protocols such as SD, EEHA, HAS, IIF, and RHC have a throughput range of 415–443 representing 85–90% of the data rate/channel capacity of the network, energy consumption in the range of 3.0–3.6 joules representing 88–95% energy efficiency of the network, effected network range of 2.98 representing 72–89% achieved improved overall performance of the network and time complexity in the range of 0.20 s representing 72–89% efficiency of the proposed SDAAA approach. Thus, our proposed SDAAA protocol outperformed the other similar types of protocols such as SD, EEHA, HAS, IIF, and RHC, from the perspective of average throughput, average energy consumption, affected network, resilience time, and time complexity in the presence of malicious nodes. In the future, we aim to extend our proposed SDAAA protocol to include a mobility model to investigate further state-of-art QoS metrics, with different data markings applicable to that situation.

## Figures and Tables

**Table 1:** Used variables and description.

Notations	Description
Me	Message
$C_{t}$	The current time of sent message
$Sig{(BS}_{n})$	Signature of Base station
h	Hash function
$k_{id}$	Sensor node’s identity
${PU}_{K}$	Public key
$C_{k_{id}}$	A certificate was issued to identify the sensor node
$C_{e}$	Certificate expiration time

**Table 2:** Simulation parameters for the proposed SDAAA protocol.

Used Parameters	Detail of Parameters
Network topology	1300 × 1300 m^2^
Total sensors	540
Node’s transmission range	40 m
Node’s sensing range	30 m
The initial energy of a node	5 joules
Bandwidth of node	50 kb/s
Simulation time/rounds	36 min
Data packet size	256 bytes
Initial pause time	15 s
Total hops	22
Proposed and contending aggregation methods	SDAAA, SD, EEHA, HAS, IIF, and RHC
Buffering capacity	50-packet buffering capacity at each node
Mobility (speed of the nodes)	0 m/s to 15 m/s
Base station location	(0, 820)
Power intensity	−14 dBm to 13 dBm.
Rx energy	13.5 mW
Tx energy	15 mW
Routing protocol	Hop-by-hop
Medium access control protocol	IEEE 802.15.4 cluster-based MAC

**Table 3:** Time complexity for SDAAA and contending SD, EEHA, HAS, IIF, and RHC protocols.

Time Complexity of Proposed Approach	Time Complexity
EEHA	*T*(*n*) = *T*(*n* − 1) + *T*(0) + *O*(*n*) =*T*(*n* − 1) + *O*(*n*) =*O*(*n*^2^)
SDAAA	$T\left(n\right)=at\left(\frac{n}{b}\right)+O\left(n\right)$ The problem consists of a finite set of inputs, but computation complexity remains constant (‘*n*’) $T\left(n\right)=t\left(\frac{n}{2}\right)+O\left(n\right)$ $T\left(n\right)=t\left(\frac{n}{2}\right)+n$ $\left(n\right)=t\left(\frac{n}{n}\right)+n$ $T\left(n\right)=t\left(1\right)+n$ $T\left(n\right)=t+n$ Where t is ignored; therefore, we obtain $T\left(n\right)=n$ *n* = *k* & *k* = *logn* By substitution, we obtain thus, the complexity is $O\left(logn\right)$
IIF	$T\left(n\right)=at\left(\frac{n}{b}\right)+O\left(n\right)$ The problem consists of a finite set of inputs, but its computation time increases linearly. Thus, $T\left(n\right)=t\left(\frac{n}{2}\right)+O\left(n\right)$ $T\left(n\right)=t\left(\frac{n}{n}\right)+O\left(n\right)$ $T\left(n\right)=t+O\left(n\right)$ Where t is ignored; therefore $T\left(n\right)=O\left(n\right)$
HAS	$T\left(n\right)=at\left(\frac{n}{b}\right)+O\left(n\right)$ Here, we divide the problem into two portions of the same size. However, the algorithm is infinite. Thus: $T\left(n\right)=2t\left(\frac{n}{2}\right)+O\left(n\right)$ $T\left(n\right)=2t\left(\frac{n}{2}\right)+O\left(n\right)$ $\left(n\right)=4t\left(\frac{n}{4}\right)+n+n$ $T\left(n\right)=4t\left(\frac{n}{n}\right)+2n$ $T\left(n\right)=4t+2n$ $T\left(n\right)=O\left(kn\right)$ $T\left(n\right)=O\left(lognn\right)$ Where *k* = *logn* $T\left(n\right)=O\left(nlogn\right)$
SD	$T\left(n\right)=at\left(\frac{n}{b}\right)+O\left(n\right)$ The problem consists of a finite set of inputs, but computation complexity remains constant (‘*n*’). $T\left(n\right)=t\left(\frac{n}{2}\right)+O\left(n\right)$ $T\left(n\right)=t\left(\frac{n}{2}\right)+n+n$ $\left(n\right)=t\left(\frac{n}{n}\right)+n+n$ $T\left(n\right)=t\left(1\right)+n+n$ $T\left(n\right)=t+n+n$ Where t is ignored; therefore, we obtain $T\left(n\right)=n+n$ *n* = *k* & *k* = *logn* we get *O*(*logn* + *n*)
RHC	$T\left(n\right)=at\left(\frac{n}{b}\right)+O\left(n\right)$ We divided the problem into two parts with different sizes according to the needs of the proposed algorithm. $T\left(n\right)=t\left(\frac{n}{3}\right)+t\left(\frac{2n}{3}\right)+O\left(n\right)$ $T\left(n\right)=t\left(\frac{n}{3}\right)+t\left(\frac{2n}{3}\right)+O\left(n\right)$ $T\left(n\right)=t\left(\frac{n}{3}\right)+t\left(\frac{2n}{3}\right)+n+n$ $T\left(n\right)=t\left(\frac{n}{n}\right)+t\left(\frac{2n}{n}\right)+n+n$ $T\left(n\right)=2t+2n$ $T\left(n\right)=lognn$ $T\left(n\right)=O\left(nlog\left(n\right)\right)$

**Table 4:** Time complexity *T*(*n*) and other characteristics of SDAAA, SD, EEHA, HAS, IIF, and RHC.

Name of Approach	Time Complexity	Impact of *T*(*n*)	Probabilistic Attack Detection	Robust to Communication Loss
SDAAA	*O*(*logn*)	Excellent	Yes	Yes
SD	*O*(*logn* + *n*)	Fair	No	Yes
EEHA	*O*(*n*^2^)	Worst	No	No
HAS	*O*(*nlogn*)	Bad	No	No
IIF	*O*(*n*)	Good	Yes	No
RHC	*O*(*nlogn*)	Bad	No	No

**Table 5:** Showing performance of SDA AA protocol comparison with the different contending approaches like SD, EEHA, HAS, IIF, and RHC protocols.

Approaches	Throughput			Average Energy Consumption			Effected Network		
	0% Malicious Node	5% Malicious Node	10% Malicious Node	0% Malicious Node	2% Malicious Node	5% Malicious Node	2% Malicious Node	5% Malicious Node	10% Malicious Node
EEHA	415 kb/s	405 kb/s	402 kb/s	3.24 Joule	3.7 Joule	4.41 joules	3.25%	4.27%	4.48%
SD	427 kb/s	407 kb/s	402 kb/s	3.4 Joule	3.82 Joule	4.42 joules	3.28%	4.89%	4.48%
HAS	436 kb/s	415 kb/s	404.4 kb/s	3.62 Joule	3.92 Joule	4.49 joules	2.98%	4.38%	4.99%
IIF	435.5 kb/s	418 kb/s	409 kb/s	3.35 Joule	3.71 Joule	3.32 joules	3.34%	4.08%	4.66%
RHC	431 kb/s	412.2 kb/s	407 kb/s	3.0 Joule	3.5 Joule	4.39 joules	3.62%	3.68%	4.47%
SDAAA	444 kb/s	426.5 kb/s	424.5 kb/s	2.6 Joule	2.91 Joule	3.32 joules	2.45%	2.62%	2.67%

## Data Availability

Available upon request.
